# Class II Division 1 malocclusion treatment with extraction of maxillary first molars: Evaluation of treatment and post‐treatment changes by the PAR Index

**DOI:** 10.1111/ocr.12412

**Published:** 2020-09-01

**Authors:** Johan W. Booij, Anne Marie Kuijpers‐Jagtman, Ewald M. Bronkhorst, Christos Livas, Yijin Ren, Mette A.R. Kuijpers, Christos Katsaros

**Affiliations:** ^1^ Private practice Gorinchem The Netherlands; ^2^ Department of Orthodontics University Medical Center Groningen Groningen The Netherlands; ^3^ Department of Orthodontics and Dentofacial Orthopedics School of Dental Medicine/Medical Faculty University of Bern Bern Switzerland; ^4^ Faculty of Dentistry Universitas Indonesia Jakarta Indonesia; ^5^ Department of Dentistry Radboud Institute for Health Sciences Radboud University Medical Center Nijmegen The Netherlands; ^6^ Dental Clinics Zwolle Zwolle The Netherlands; ^7^ Department of Orthodontics and W.J. Kolff Institute for Biomedical Engineering and Materials Science University Medical Center Groningen Groningen The Netherlands; ^8^ Department of Dentistry ‐ Orthodontics and Craniofacial Biology Radboud University Medical Center Nijmegen The Netherlands

**Keywords:** angle class II, longitudinal study, maxillary first molar extraction, PAR index, treatment outcome

## Abstract

**Objective:**

To investigate occlusal result and post‐treatment changes after orthodontic extraction of maxillary first permanent molars in patients with a Class II division 1 malocclusion.

**Setting and Sample:**

Retrospective longitudinal study in a private practice, with outcome evaluation by an independent academic hospital. Ninety‐six patients (53 males, 43 females) consecutively treated by one orthodontist with maxillary first permanent molar extraction were studied, divided into three facial types, based on pre‐treatment cephalometric values: hypodivergent (n = 18), normodivergent (n = 21) and hyperdivergent (n = 57).

**Methods:**

Occlusal outcome was scored on dental casts at T1 (pre‐treatment), T2 (post‐treatment) and T3 (mean follow‐up 2.5 ± 0.9 years) using the weighted Peer Assessment Rating (PAR) Index. The paired sample *t* test and one‐way ANOVA followed by Tukey's post hoc test were used for statistical analysis.

**Results:**

PAR was reduced by 95.7% and 89.9% at T2 and T3, respectively, compared with the start of treatment. The largest post‐treatment changes were found for overjet and buccal occlusion. Linear regression analysis did not reveal a clear effect (R‐Square 0.074) of age, sex, PAR score at T1, incremental PAR score T2‐T1, overjet and overbite at T1, and facial type on the changes after treatment (incremental PAR score T3‐T2).

**Conclusions:**

The occlusal outcome achieved after Class II division 1 treatment with maxillary first permanent molar extractions was maintained to a large extent over a mean post‐treatment follow‐up of 2.5 years. Limited changes after treatment were found, for which no risk factors could be discerned.

## 1 INTRODUCTION

A great variety of treatment options exists for the treatment of Class II malocclusions, including facial orthopaedic, functional, non‐extraction and extraction procedures. Treatment options depend on the type and severity of the malocclusion, age and facial growth status of the patient, educational background of the orthodontist and treatment preferences of the patient.^1^ In young patients, growth modification with functional appliances or extra‐oral traction is often the treatment of choice, although nowadays the concept of long‐term growth modification of the mandible and maxilla is questioned.^2^, ^3^ Recent alternatives for Class II correction by upper molar distalizing mechanics are provided by appliances fixed with temporary anchorage devices (TAD’s), an implant in the frontal part of the palate, or bone anchors attached to the zygomatic arches.^4^ When crowding in the upper and lower arch is present, orthodontists may choose a dental correction by extraction of four premolars. When there is a good lower arch in the presence of an overjet, extractions may be limited to upper first or second premolars only. There are also other options, such as extraction of the maxillary second or first molars.

Williams^5^ was in 1979 one of the first to publish a treatment concept involving extraction of maxillary first molars using a light‐wire technique. In 2009, the method was described in detail by Booij et al^6^ Several studies have reported the short‐term results of this procedure.^7^, ^8^, ^9^ Using the PAR index to measure occlusal outcome in 100 consecutive patients at the end of active treatment, 73% were in the 'greatly improved' and 27% in the 'improved' group.^7^ There were no patients in the 'worse or no different' group. The cephalometric analysis revealed that this type of treatment had only a minimal bite‐closing effect, while no significant differences for change in mandibular plane angle were found between different facial types. The patients showed a flattening of the profile and an increase in the nasolabial angle, comparable to the soft‐tissue outcomes of other extraction modalities, as reported in systematic reviews.^10^, ^11^

To our knowledge, no previous studies have reported on stability of Class II division 1 malocclusion treatment with maxillary first permanent molar extractions. As post‐treatment changes occur mostly in the first 2 years,^12^, ^13^, ^14^ the aim of this study was to evaluate occlusal results of Class II division 1 treatment with extraction of maxillary first permanent molars after a mean follow‐up period of 2.5 years, in a large group of consecutively treated patients.

## 2 SUBJECTS AND METHODS

### 2.1 Subjects

This was a retrospective, longitudinal, one‐group outcome study in a private practice, with outcome evaluation by an independent academic hospital. The research was conducted in accordance with the Helsinki Declaration with regard to research on human subjects. All parents and patients agreed to have their patient records used in the study and gave signed informed consent. Ethical approval was not needed, as this was an observational study using anonymized, routinely collected health data.

The sample consisted of 99 consecutively treated patients (45 girls, 54 boys) treated by 1 orthodontist (JWB). The following inclusion criteria were used: Caucasian, Class II division 1, sagittal overjet of ≥4 mm, extraction of maxillary first permanent molars, no missing teeth or agenesis, maxillary third molars present, and one‐stage full fixed appliance treatment. Cleft lip and palate patients and patients with craniofacial deformities were excluded. The intake period was from December 1997 to August 2002.

Dental casts of all patients were made at T1 (pre‐treatment), T2 (post‐treatment) and T3 (follow‐up). The standard recall schedule was 2 years after treatment, and the minimum follow‐up was set at 24 months with a deviation of −20%. To study the effect of treatment for different facial types, the sample was divided into three groups, based on pre‐treatment cephalometric values: horizontal (ANS‐Me/N‐Me ≤56%; n = 18), normal (56% <ANS‐Me/N‐Me <58%; n = 21) and vertical (ANS‐Me/N‐Me ≥58%; n = 60).^7^, ^15^

### 2.2 Treatment method

Treatment with fixed appliances started 2 weeks after the extraction of the maxillary first permanent molars. In case of a deep bite, the extractions were delayed, with initial placement of an upper bite plate and a fixed appliance in the lower arch. Second maxillary molars were fully erupted before the extractions were carried out. All patients were treated with fixed appliances according to the principles of the light‐wire technique. In short, at the start of treatment in the Class II correction phase, horizontal elastics (Light 5/16,TP, Westville, USA) were attached from a high hat lock pin in the upper canine bracket to a ball end hook on the upper second molar band. The patient was instructed to replace these elastics once a week. Class II elastics (Medium 5/16,TP, Westville, USA) were used and had to be replaced every day. Wearing time was reduced as soon as a solid Class I premolar occlusion was reached. After appliance removal, fixed retainers were bonded to all upper and lower anterior teeth (0.195‐inch Wildcat, GAC, Central Islip, NY, US). In cases of non‐occlusion of the mandibular second molars, a buccal retention wire (0.195‐inch Wildcat, GAC, Central Islip, NY, US) was bonded between the first and second molar to keep these teeth in position. These buccal retention wires were removed after complete eruption of the maxillary third molars.

### 2.3 Occlusal outcome

For assessment of the results, the dental casts were randomly placed on a table and identified by only a non‐traceable number. The scoring was performed by one observer (CL) calibrated in the use of the Peer Assessment Rating (PAR) Index, who was not involved in the treatment.

Occlusal outcome was scored on the dental casts at T1 (pre‐treatment), T2 (post‐treatment) and T3 (2 or more years post‐treatment) using the PAR Index.^16^ The PAR Index consists of the sum of seven subcomponent scores: upper anterior segment, lower anterior segment, left and right buccal occlusion, overjet, overbite and centre line. Weighted PAR scores (British weightings) were used, which means that the individual scores for overjet were multiplied by 6, overbite by 2 and centre line by 4. A weighted PAR score of 0 means good alignment and higher scores indicate the level of irregularity. The degree of success of the orthodontic treatment is reflected by the percentage reduction in the total weighted PAR score. The PAR subcomponent 'anterior cross bite' was excluded because this sample consisted of Class II division 1 patients and only one patient scored on this item. It concerned an end‐to‐end position of two lateral incisors (patient number 65). Nomograms were used to visualize the degree of improvement following treatment and to visualize the degree of final improvement between the starting condition and 2 years post‐treatment. In these nomograms,^17^ the degree of change of the weighted PAR score is divided into three categories: worse or no different (cases with less than 30% reduction), improved (patients with ≥30% reduction) and greatly improved (generally a reduction of 22 weighted PAR points or more).

The weighted PAR scores were used to evaluate treatment outcome, treatment efficiency, operator experience and the change after treatment. Treatment efficiency was defined as the treatment efficiency index (TEI) according to Janson et al^18^ as the PAR reduction between T1 and T2 divided by treatment duration (in months). Furthermore, the weighted PAR scores of the three vertical facial types were compared.

To determine the error of the method, the same observer re‐assessed 21 series of models (at T1, T2 and T3) 2 weeks after the first assessment.

The eruption status of the third molars at T3 was evaluated on the dental casts and the radiographs (orthopantomogram and/or lateral cephalogram).

### 2.4 Statistics

The statistical analysis was performed using SPSS version 22 for Windows (IBM, North Castle, USA). For the overall PAR score, the reliability coefficients between the two measurements were calculated as Pearson's correlation coefficients. Paired sample t tests were applied to identify systematic differences between the first and second measurement. The duplicate measurement error (DME) was calculated as the SD of the difference between two observations divided by √2. The intra‐observer reliability for the PAR subcomponents was calculated using weighted kappa statistics. A kappa less than 0 reflects 'poor', 0 to 0.20 'slight', 0.21 to 0.4 'fair', 0.41 to 0.60 'moderate', 0.61 to 0.8 'substantial' and above 0.81 'almost perfect' agreement.^19^

Outcomes are presented as a variable with a mean and ±SD. The paired sample *t* test was applied to analyse the changes in the PAR score between T1 and T2, T2 and T3, and T1 and T3.

One‐way ANOVA followed by Tukey's post hoc test was applied to test for differences in the TEI between the three facial types.

Linear regression analysis was applied to analyse the effects of the independent variables age, sex, PAR score at T1, incremental PAR score T2‐T1, overjet and overbite at T1, and facial type at T1 on the incremental PAR score T3‐T2 (the dependent variable).

A *P* value of <.05 was considered to indicate statistical significance.

## 3 RESULTS

### 3.1 Subjects

All 99 patients finished their treatment, and no treatments were discontinued or finished early. Two patients (2.02%) were lost to follow‐up at T3, and one patient had a follow‐up period of less than the target follow‐up and was excluded, leaving a final sample size of 96 (53 boys and 43 girls). A hypodivergent facial type was seen in 18 patients (14 boys, 4 girls), 21 patients were normodivergent (10 boys, 11 girls), and 57 were hyperdivergent (29 boys, 28 girls). The average treatment duration was 2.5 years (SD 0.6; range 1.4‐4.5).

The mean age at the start of treatment was 13.2 years (SD 1.5; range 10.5‐17.2), the mean age at T2 was 15.7 years (SD 1.6; range 12.4‐19.8), and the mean age at T3 was 18.2 years (SD 1.8; range 14.4‐23.9). The average post‐treatment period was 2.5 years (SD 0.9; range 1.7‐5.8).

### 3.2 Error of the method

For the overall PAR score, the Pearson's correlation coefficient was 0.998. The duplicate measurement error (DME) was 0.638 PAR points. The mean difference between the first and second measurement was 0.159 PAR points (95% CI, −0.068…0.386) which was not statistically significant (*P* = .167). The kappa values for the weighted subcomponents ranged from 0.833 to 1.000, reflecting almost perfect agreement.

### 3.3 Outcome

**FIGURE 1:**
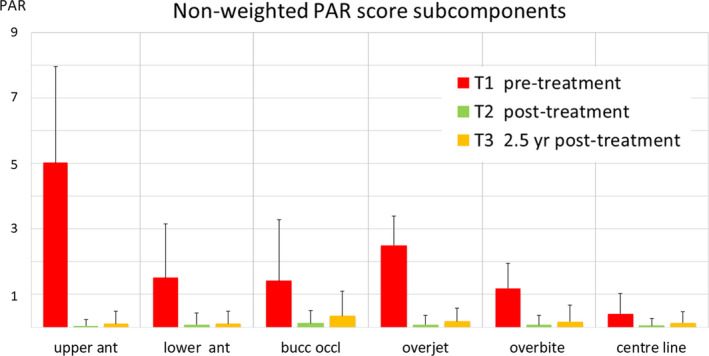
Non‐weighted PAR scores (mean and SD) for the subcomponents of the PAR before treatment, after treatment and after a mean follow‐up of 2.5 years after treatment [Colour figure can be viewed at www.wileyonlinelibrary.com]

**FIGURE 2:**
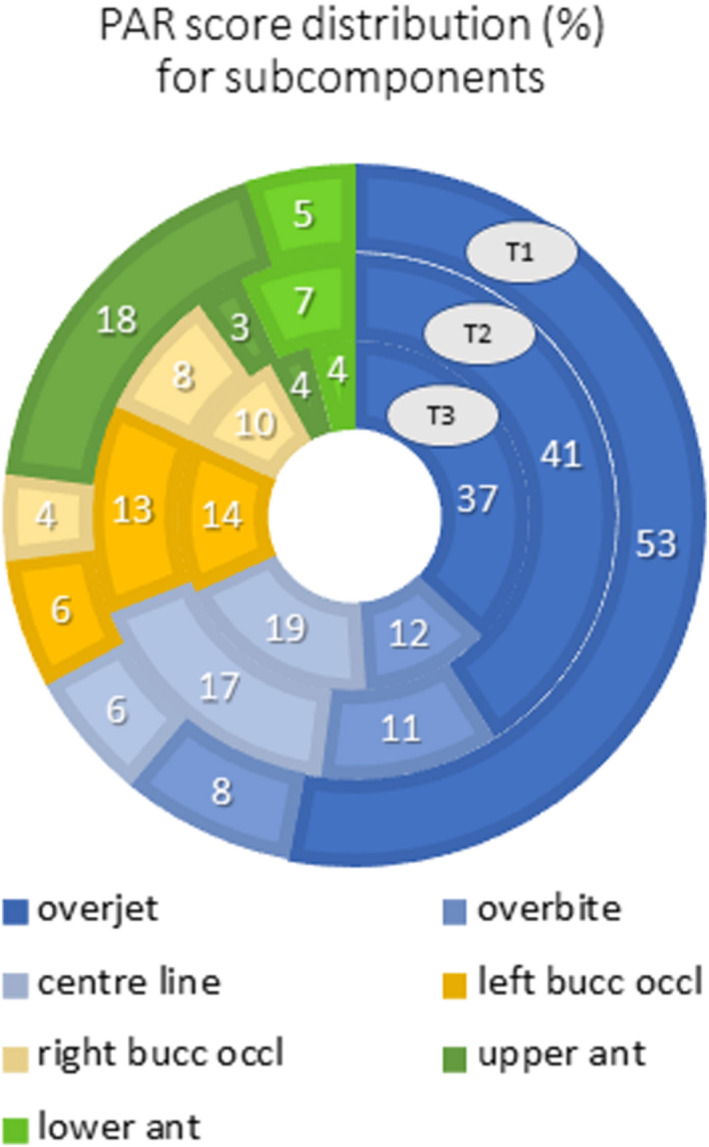
Ring map of the distribution of the PAR subcomponents before treatment, after treatment and after a mean follow‐up of 2.5 years after treatment [Colour figure can be viewed at www.wileyonlinelibrary.com]

The results for the PAR index at T1, T2 and T3 are presented in Table 1 and Figure 1. The mean weighted PAR score at the start of treatment (T1) was 28.26 (SD 7.10). At the end of treatment (T2), the PAR was 1.22 (SD 2.36), and this rose slightly to 2.86 (SD 3.57) during the follow‐up period (T2‐T3). The largest changes after treatment were found for overjet and buccal occlusion. PAR was reduced by 95.7% at the end of treatment and was still reduced by 89.9% at the end of the follow‐up period as compared with the start of treatment. The overjet, overbite and centre line—the three PAR subcomponents that have a weighting—represented almost the same percentage of the total PAR score at T1, T2 and T3, being 67%, 69% and 68%, respectively. This is demonstrated by the blue surfaces of the three subcomponents in the ring map (Figure 2). In addition to this, the contribution of the other subcomponents changed from T1 to T3. For example, at the end of the follow‐up period the left and right buccal occlusion represented 24% of the total PAR score while this was 10% at T1.

**TABLE 1:** Non‐weighted PAR scores (mean and SD) for the subcomponents of the PAR before treatment, after treatment, and after a mean follow‐up of 2.5 years after treatment

Time	N	PAR Total (weighted)	PAR subcomponents						
			Upper anterior segment	Lower anterior segment	Right buccal occlusion	Left buccal occlusion	Overjet	Overbite	Centre line
T1	96	28.26 (7.10)	5.02 (2.94)	1.51 (1.64)	1.24 (1.11)	1.59 (2.61)	15.00 (5.37)	2.38 (1.52)	1.63 (2.43)
T2	96	1.22 (2.36)	0.04 (0.20)	0.08 (0.35)	0.10 (0.34)	0.16 (0.42)	0.50 (1.67)	0.13 (0.57)	0.21 (0.89)
T3	96	2.86 (3.57)	0.11 (0.38)	0.11 (0.38)	0.30 (0.73)	0.40 (0.79)	1.06 (2.46)	0.33 (0.99)	0.54 (1.38)

**FIGURE 3:**
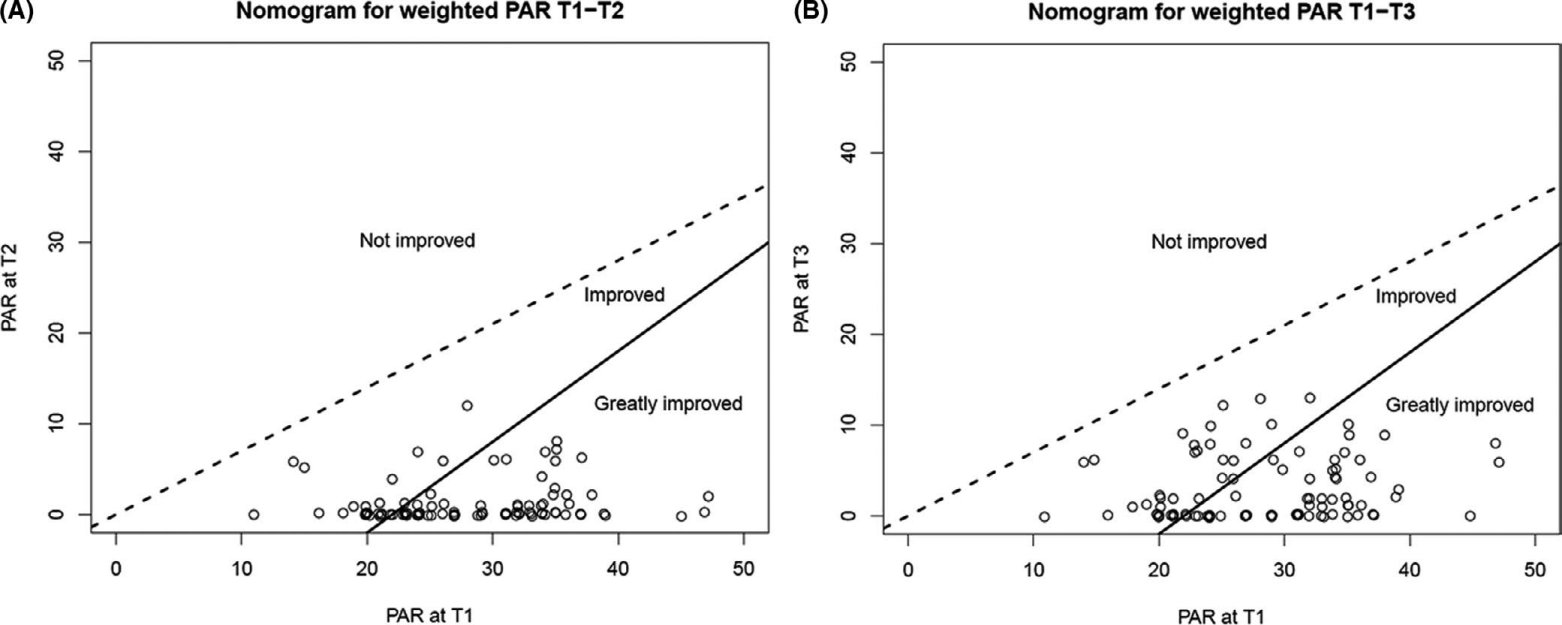
Nomograms showing the categorization of improvement of the weighted PAR scores at T1 plotted against T2 (A), and T1 plotted against T3 (B)

Figure 3A shows the nomogram with the weighted PAR score at T1 compared with the score at T2. No patients were in the 'not improved' section, 26 patients (27.1%) were in the 'improved' section, and 70 patients (72.9%) were in the 'greatly improved' section. Figure 3B shows the nomogram with the weighted PAR score at T1 compared with the score at T3. No patients were in the 'not improved' section, 37 patients (38.6%) were in the 'improved' section, and 59 patients (61.4%) were in the 'greatly improved' section.

Table 2 shows the changes in the PAR index and the changes of the subcomponents during treatment (T2‐T1), after treatment (T3‐T2) and for the entire time period (T3‐T1). At the end of treatment, a significant decrease of –27.04 (95% CI: −28.51…−25.57) PAR points was found (*P* < .001). The scores for all subcomponents also decreased significantly during treatment. After treatment (T3‐T2), there was a slight but significant increase in the PAR index of 1.65 PAR points (95% CI: 0.99…2.30; *P* < .001). The scores for all subcomponents increased significantly, except for the lower anterior segment.

**TABLE 2:** Changes of the weighted PAR scores (means and SD) and the subcomponents during treatment (T2‐T1), after treatment (T3‐T2) and for the entire time period (T3‐T1). Results of paired samples *t* test

Time period (N)	Weighted PAR total and subcomponents	Paired differences			Sign. (2‐tailed)
		Mean	95% Confidence interval of the difference		
			Lower	Upper	
T2‐T1 (N = 96)	PAR total	−27.04	−28.51	−25.57	<0.001
	upper anterior segment	−4.98	−5.58	−4.38	<0.001
	lower anterior segment	−1.43	−1.76	−1.09	<0.001
	right buccal occlusion	−1.14	−1.36	−0.91	<0.001
	left buccal occlusion	−1.44	−1.97	−0.90	<0.001
	overjet	−14.5	−15.61	−13.39	<0.001
	Overbite	−2.25	−2.57	−1.93	<0.001
	centre line	−1.42	−1.93	−0.90	<0.001
T3‐T2 (N = 96)	PAR total	1.65	0.99	2.30	<0.001
	upper anterior segment	0.07	0.01	0.14	0.034
	lower anterior segment	0.03	0.00	0.07	0.083
	right buccal occlusion	0.20	0.08	0.31	0.001
	left buccal occlusion	0.24	0.11	0.37	<0.001
	overjet	0.56	0.00	1.12	0.049
	overbite	0.21	0.02	0.40	0.032
	centre line	0.33	0.05	0.61	0.02
T3‐T1 (N = 96)	PAR total	−25.40	−26.90	−23.89	<0.001
	upper anterior segment	−4.91	−5.51	−4.30	<0.001
	lower anterior segment	−1.40	−1.74	−1.05	<0.001
	right buccal occlusion	−0.94	−1.17	−0.70	<0.001
	left buccal occlusion	−1.20	−1.73	−0.66	<0.001
	overjet	−13.94	−15.09	−12.79	<0.001
	Overbite (N = 95)	−2.04	−2.39	−1.69	<0.001
	centre line	−1.08	−1.64	−0.53	<0.001

Linear regression analysis for the effect of the independent variables age, sex, PAR score at T1, incremental PAR score T2‐T1, overjet and overbite at T1, and facial type on the changes after treatment (incremental PAR score T3‐T2) revealed only a minor effect of the change of the total PAR score during treatment on the changes after treatment (B = −0.291, 95% CI −5.581…−0.001, *P* = .049; R‐Square .074).

### 3.4 Operator experience

To determine the operator experience, the first 20 treated patients were compared with the last 20 treated patients with regard to the weighted PAR score at T2. For the first 20 patients, the weighted PAR score at T1 was 28.4 (SD 7.1), and for the last 20 patients, it was 26.6 (SD 7.1). The changes in the PAR scores for the first and last 20 patients between T1 and T2 were −26.95 (SD 6.70) and −25.95 (SD 7.62), respectively, and not significantly different from each other (independent samples *t* test *P* = .662). Operator experience had also no effect on the change in the PAR score between T2 and T3, which amounted to 1.6 (SD 3.82) PAR points for the first 20 patients and 1.2 (SD 1.88) for the last 20 patients (*P* = .677). In both groups, 8 of 20 patients (40%) showed a change in the PAR score after treatment.

The Treatment Efficiency Index (TEI) for the total group was 3.35 (SD 0.85). We also compared TEI for the first and last 20 treated patients between T1 and T2. The first group had a TEI of 2.88 (SD 0.65), and the more recently treated group had a TEI of 3.87 (SD 0.70). This difference was highly significant (*P* < .001), indicating a greater PAR reduction per treatment month in the more recently treated group.

### 3.5 Maxillary third molars

In 52 patients (54.2%), a buccal retention wire was placed at the first and second lower molar in cases of non‐occlusion of the mandibular second molars at T2 (n = 4 on one side, n = 48 at two sides). At T3 in 11 patients (11.5%), these wires were still present (n = 4 on one side, n = 7 at two sides). In 80 patients (83.3%), both maxillary third molars were present at T3. In 8 patients, one of the molars was erupted at that point of time. The 24 as yet unerupted maxillary third molars were checked on the X‐rays, and 23 of them had a good prognosis for eruption. The prognosis of 1 molar was doubtful.

## 4 DISCUSSION

We investigated occlusal outcome of Class II division 1 treatment after extraction of the upper first permanent molars in a group of 97 patients. This retrospective longitudinal study addresses an interesting topic for clinical orthodontists as it reports the results of a large consecutively treated cohort of relatively rare material.

At the end of treatment, the mean PAR score showed an improvement of 95.7%. The PAR score changed 5.8% after treatment, resulting in an improvement of the PAR score of 89.9% after a mean follow‐up period of 2.5 years. A PAR score improvement of 80% at the end of active treatment is accepted as a 'good result', while a good standard of orthodontic treatment is achieved when the reduction of the PAR score is greater than 70%.^20^ Studies reporting post‐treatment outcomes using the PAR index in which the patient group was restricted to Class II patients are limited. In a study on 50 Class II division 1 malocclusions, Otuyemi and Jones^21^ found a post‐treatment PAR score improvement of 82.5%, which decreased after one year to 69.9%, and 10 years post‐treatment 48.6% of the improvement persisted. Late lower anterior crowding was the major factor for this deterioration. In our sample, upper‐ and lower‐bonded 3‐3 retainers were part of the treatment protocol, explaining the minimal change in the anterior segments in our patient group.

Except for Class III malocclusions, nearly all studies on long‐term treatment outcomes have not been restricted to specific malocclusion types.^22^, ^23^, ^24^, ^25^ All those studies had varying lengths of follow‐up, and all reported a decrease of the PAR score after treatment, varying from 12.9% to 33%. Al Yami et al^12^ studying a large group of patients reported a post‐treatment PAR score improvement of 67.1%, which decreased 2 years after the end of retention to 54%. This reduction continued at a slower pace, and 10 years post‐treatment the PAR improvement was 45.2%. Al Yami et al^12^ concluded that 50% of the post‐treatment change occurs in the first 2 years after treatment. The present study's outcome of 5.8% post‐treatment change, after a mean follow‐up of 2.5 years, is very acceptable.

The subcomponents that changed the most after treatment were overjet and left and right buccal occlusion, as may be expected because this was a sample of Class II division 1 malocclusion. The rating for the buccal occlusion is very sensitive, and in our sample, the occlusal relationships between the upper and lower first molar at T1, and upper second molar and lower first molar at T2 and T3, were assessed. The anatomy of the upper second molar is slightly different from that of the upper first molar, and this influences the PAR score for the buccal occlusion subcomponent in a negative way.

In only a few studies that used the PAR index to assess treatment outcome has the course of the individual unweighted subcomponents been reported. Miao and Liu^24^ found a large relapse for alignment, overjet and overbite. Al Yami et al^12^ concluded, after a 10‐year follow‐up study, that all subcomponents changed gradually over time but remained stable from 5 years post‐retention on, except the lower anterior component in cases without a fixed retainer. In an 8‐year follow‐up study,^25^ a 3‐fold increase in the irregularity of the lower anterior teeth was found in participants without a lower cuspid‐to‐cuspid retainer. All patients in our study had fixed retention in the upper and lower arch from canine to canine, bonded to all teeth. This explains the minimal change we found in the post‐treatment period for the upper and lower anterior subcomponents. The recent study of Schütz‐Fransson et al^26^ showed that maintaining fixed retainers from canine to canine, for 2 to 3 years only, cannot prevent changes of mandibular incisor alignment later on, and therefore, lifelong retainers are needed if the patient wants to keep the lower front teeth straight. In our study, the post‐treatment changes of the other subcomponents were small as well. This may be explained by the fact it concerns a one‐phase treatment aimed at tooth movement rather than growth modification.

The regression analysis showed that change after treatment (with the incremental PAR score T3‐T2 as the dependent variable) could only be explained for 7.4% from the independent variables we tested: age, sex, PAR score at T1, incremental PAR score T2‐T1, overjet and overbite at T1, and facial type, which means we could not find clear risk factors for change in the PAR score after treatment. Some studies gave similar outcomes,^22^, ^23^ while others showed that patients with more severe PAR index scores at the start of treatment tended to be less stable^27^ and that females had more changes than males 10 and 15 years post‐treatment.^28^ A systematic review on long‐term stability after orthodontic treatment^29^ was not able to draw evidence‐based conclusions regarding stability in Class II patients due to the low quality of the available studies. However, the number of published orthodontic randomized controlled trials is gradually increasing.^30^ It is to be hoped that this improvement in study design will provide more insight into factors related to treatment stability, resulting in better predictability of the long‐term stability of any individual orthodontic treatment.

Concerning operator experience, there was no difference in treatment outcome for the first 20 patients and the last 20 patients of our study group. All patients were treated by one experienced orthodontist (JWB) who apparently did not change his standard of case finishing over the years. We could not verify this with other studies, as such a finding has not been reported before now. On the other hand, the Treatment Efficiency Index (TEI) gave a highly significant difference between the first 20 and the last 20 patients, indicating a greater PAR reduction per treatment month in the more recently treated group (TEI = 2.88 vs TEI = 3.87), in turn indicating that the orthodontist became more efficient with experience. The total group had a mean TEI of 3.35 (SD 0.85). Janson et al^18^ found a TEI of 3.78 (SD 1.27) for 69 Class II patients treated by a 2‐maxillary‐premolar‐extraction protocol. In a similar study of 26 patients, Pinzan‐Vercelino et al^31^ reported a TEI of 4.02 (SD 1.37). Comparable findings for upper first molar extractions are not available.

This is the first study on post‐treatment changes of Class II division 1 treatment including extraction of maxillary first permanent molars in which a large patient group was involved. The favourable results reported here will support the orthodontist in the clinical decision whether to extract these molars or not. Large restorations, hypomineralization or endodontic treatment of these teeth will make it easier to decide for extraction. In children older than 11 years, the maxillary first permanent molar is the most caries‐prone tooth.^32^ Furthermore, in Dutch children an increase in the prevalence of molar‐incisor hypomineralization was found between 1999 and 2003,^33^, ^34^ when 12.7% of the children had at least two affected molars. It should also be noted that endodontically treated first permanent molars have more complications than other endodontically treated single‐rooted teeth.^35^ To optimize the quality of the dentition, extraction of poorly conditioned maxillary first permanent molars is a good option for Class II malocclusion treatment, if the second and third molars are of good quality. The third molars have also been shown to have a better prognosis for normal eruption when the first molars are extracted^8^ which was also confirmed in the present study as only one third molar showed a bad prognosis and 83.3% of the third molars had already erupted at the follow‐up observation.

### 4.1 Limitations

This study was a longitudinal cohort study for which the data were collected in a private practice. All treatments were carried out by the same orthodontist. As compared to a multicentre, multi‐operator trial, the single‐centre, one‐operator study design is less favourable for the generalizability of the results. As compared to the randomized controlled trial, the retrospective study design has well‐known drawbacks. Not all clinical orthodontic research questions can be studied, however, in randomized controlled trials. For example, ethical concerns—extraction version non‐extraction therapy—may limit the application of the most rigorous design.

As this was a one‐group longitudinal study, we are not able to determine the contribution of physiological changes to the treatment and post‐treatment changes we found. An earlier study, however, showed that the PAR score in non‐treated individuals between 12 and 22 years of age remained the same, irrespective of the Angle classification, although clinically relevant changes were found in individual cases.^36^

We are aware of the limitations of the PAR index. Dental variables, like proclined lower incisors and retroclined upper incisors, are not represented in the rating. Furthermore, the PAR index uses a weighting system for several subcomponents of the index. Overjet, for example, has a weighting of 6 in the British weighting system, which adds considerably to a high pre‐treatment PAR score in a sample of Class II division 1 cases with a large overjet, as in our study. For that reason, it is easier to realize remarkable changes in the PAR score when the initial PAR score is high.^37^ A recent study in a Chinese population suggested that different Angle classifications may need different weightings.^38^ This supports the discussion, for more than two decades, of the British weightings overemphasizing overjet and insufficiently weighting overbite.^39^ Some have suggested extending the PAR index with a score for sufficient torque, good axial control of lower incisors and the irregularity index.^27^, ^40^

Overall quality of the treatment manifested as the presence of root resorption, gingival recessions, white spots, dysfunctions, facial aesthetics, and patient satisfaction and quality of life were not measured in this study.

## 5 CONCLUSION

The occlusal outcome achieved after Class II division 1 treatment with maxillary first permanent molar extractions was maintained to a large extent over a mean post‐treatment follow‐up of 2.5 years. Sex, age, facial type, overbite and overjet, and the PAR score at the start of treatment had no effect on the changes after treatment.
